# Immunopathogenesis of Toxoplasmosis in Pregnancy

**DOI:** 10.1155/S1064744997000197

**Published:** 1997

**Authors:** Jean Dupouy-Camet

**Affiliations:** Laboratoire de Parasitologie-Mycologie UFR Cochin Port Royal 27 rue du Faubourg St Jacques Paris 75014 France

## Abstract

The immunopathogenesis of toxoplasmosis during pregnancy is not completely understood. This paper will try to discuss the most frequently asked questions about the immunopathogeny of congenital toxoplasmosis: differential virulence of *Toxoplasma* isolates, genetic susceptibility to infection, facilitation of placental transfer, models of congenital toxoplasmosis, and transmission in seropositive hosts. Most published data suggest a role of the genetic background of the host and of the parasite. Models of congenital toxoplasmosis have been evaluated, but it appears that the conclusion drawn would be barely appropriate to understand the pathogenesis in pregnant women.


Infectious Diseases in Obstetrics and Gynecology 5:121–127 (1997)
© 1997 Wiley-Liss, Inc.

### Immunopathogenesis of Toxoplasmosis in Pregnancy

Jean Dupouy-Camet
*Laboratoire de Parasitologie-Mycologie, UFR Cochin Port Royal, Paris, France*

ABSTRACT

**The immunopathogenesis of toxoplasmosis during pregnancy is not completely understood. This paper will try to discuss the most frequently asked questions about the immunopathogeny of congenital toxoplasmosis: differential virulence of *Toxoplasma* isolates, genetic susceptibility to infection, facilitation of placental transfer, models of congenital toxoplasmosis, and transmission in seropositive hosts. Most published data suggest a role of the genetic background of the host and of the parasite. Models of congenital toxoplasmosis have been evaluated, but it appears that the conclusion drawn would be barely appropriate to understand the pathogenesis in pregnant women. Infect. Dis. Obstet. Gynecol. 5:121–127, 1997.** © 1997 Wiley-Liss, Inc.

KEY WORDS
*Toxoplasma gondii*, immune response, pregnancy, strains, congenital

Toxoplasmosis is a parasitic infection caused by the protozoan *Toxoplasma gondii* that, when contracted by a pregnant woman, can pose a serious risk to her unborn baby. Fortunately, a pregnant woman can follow some simple precautions that can reduce her risk of infection. A pregnant woman who contracts toxoplasmosis for the first time during pregnancy has about a 30 percent chance of passing the infection on to her fetus.[1] However, the risk and severity of the baby's infection depend partly on the timing of the mother's infection. Studies suggest that, when mothers are infected in the first trimester, 14 percent of fetuses become infected, as compared to 29 percent in the second trimester and 59 percent in the third.[2] Babies whose mothers had toxoplasmosis in the first trimester usually have the most severe infections. If no screening program is carried out, most infected babies appear normal at birth. However, most of them will develop sight-threatening eye infections months to years after birth. Some also will develop hydrocephalus, mental retardation, learning disabilities or seizures. Toxoplasmosis during pregnancy also can result in miscarriage or stillbirth. Other infected babies have a severe *Toxoplasma* infection that is evident at birth with severe eye infections, hepatosplenomegaly, icterus and hydrocephalus.[3]

The parasite is most often picked up through exposure to cat feces or by eating raw or undercooked meat that is contaminated with the parasite. Cats often become infected when they eat an infected rodent or bird. The parasite reproduces in the cat's intestine, and a form of the parasite (oocysts) ends up in the cat's litter box, sand or soil. This form of the parasite becomes infectious within days and is resistant to most disinfectants. Under the right temperature and humidity conditions, the parasite may live in soil for more than a year. Infected cats usually appear healthy.

Toxoplasmosis is one of the most common infections in the world as proven by the high prevalence of antibodies in the adult population, although there are important geographic discrepancies.[1] Most cases of toxoplasmosis are undiagnosed. Symptoms, if any, tend to resemble flu. Active infection normally occurs only once in a lifetime. Although the parasite remains in the body indefinitely, it is generally harmless and inactive unless the immune system is not functioning properly. If

Correspondence to: J. Dupouy-Camet, Laboratoire de Parasitologie-Mycologie, UFR Cochin Port Royal, 27 rue du Faubourg St Jacques, 75014, Paris, France, E-mail: dupouyca @ imaginet.fr

Received 1 October 1997

Accepted 21 October 1997

a woman develops immunity to the infection before pregnancy, there is rarely any danger of passing it on to her baby (see general information at http://babynet.ddwi.com/tlc/pregnancy/toxoplas.html).

If basic knowledge concerning the biology of this parasite or the immune response in experimental animals is important, the knowledge on the pathogenesis of congenital toxoplasmosis is relatively scarce. This paper will try to discuss the most frequently asked questions about the immunopathogeny of toxoplasmosis in pregnancy.

### ARE SOME ISOLATES OF *TOXOPLASMA GONDII* MORE VIRULENT?

*Toxoplasma gondii* was discovered at the beginning of the century in North Africa and is considered as the only representative of the genus *Toxoplasma*.

### Parasitic Stages

Three stages of the parasite can be found in infected mammals: the sporozoite, the tachyzoite and the bradyzoite. Sporozoites are liberated by the digestion of infective oocysts (result of the sexual multiplication of the parasite in the cat intestine) and will penetrate through the intestinal epithelium to infect host cells. The tachyzoite is a rapidly intracellular dividing form responsible for the acute phase of the disease and able to penetrate in practically all types of cells. The host immune response will lead to the transformation of the tachyzoites in bradyzoites. This conversion could depend on environmental triggers such as pH, temperature via induction of heat-shock proteins, anti-mitochondrial drugs, nitric oxide and TNF-alpha.[4] The bradyzoite is a slowly dividing form giving cerebral or muscular cysts and responsible for a life-long immune stimulation of the infected host. Hosts can be infected by these three parasitic stages: oocysts by contact with contaminated cat feces, tachyzoites by transplacental contamination, bradyzoites by ingestion of raw meat-containing cysts. The virulence of these stages should be similar but antigenic differences have been described.[5,6]

### Parasitic Strains

One of the characteristics of many *Toxoplasma* strains is their variation in virulence. For example the highly virulent RH strains will kill experimentally infected mice in less than a week, whereas most strains isolated from congenital cases of toxoplasmosis or animals will give a chronic disease in mice characterised by the presence of cysts in the brain. There is no correspondence between virulence in humans and virulence in mice. Analysis of different strains by isoenzymes allowed the description of at least five zymodemes.[7] Isolates highly pathogenic for mice (e.g., RH strain) belong to zymodeme 1 or 5. Isolates giving a chronic infection in mice belong to zymodeme 2 (e.g., Prugnaud, 76K, Me49, Beverley), zymodeme 3 (e.g., C56) or zymodeme 4. Isolates from human congenital toxoplasmosis belonged to the five groups.[7,8] More recent data with restriction fragment length polymorphism indicate that *Toxoplasma gondii* consists of three clonal lineages, designated I, II and III. Group I corresponds to zymodeme 1, group II to zymodemes 2 and 4, group III to zymodeme 3. More than 70% of human disease cases are associated with type II strains.[9] The type II strain would also be the most prevalent in food animals such as pigs and sheep. Acute virulence in mice is strictly observed in type I strains, indicating that a genetic determinant(s) unique to this lineage controls acute pathogenesis.[10,11]

### Strains and Congenital Infections

No link is proven between a particular type of strain and transplacental passage. However, in an experimental model in Fisher rats, Zenner et al.[12] using three different strains (RH, 76K & Prugniaud) obtained significant differences of congenital toxoplasmosis between the two strains RH and Prugniaud and strain 76K. The rates of infected fetuses were 58.2% for RH, 62.8% for Prugniaud and 35.2% for 76k, respectively. However, though having apparently different rates of placental transmission, strains 76K and Prugniaud belong to the same zymodeme. These different types of virulence could be linked to different surface receptors or antigens whose role in pathogenesis is not fully elucidated.

### ARE SOME INDIVIDUALS MORE SENSITIVE TO INFECTION?

It is well proven that different mouse strains exhibit different levels of resistance to *Toxoplasma gondii*.

### Genetic Control

Genetics of survival after oral *Toxoplasma gondii* infection were studied by using recombinant inbred strains of mice derived from resistant A/J (A) and susceptible C57BL/6J (B) progenitors, F1 progeny of crosses between A/J and C57BL/6J mice, and congenic mice (B10 background). Analysis of the pattern of survival indicated that survival was regulated by a minimum of five genes. One of these genes appears to be linked to the H-2 complex.[13,14] BALB/c mice infected intraperitoneally will have fewer brain cysts than C57/BL mice (15); B10 mice (H-2b) are cyst susceptible and B10.A mice (H-2a) are cyst resistant,[16] confirming that susceptibility to *Toxoplasma gondii* and the number of brain cysts in mice are affected by genes linked to the H-2 locus. In a study by Deckert-Schluter et al.,[17] the influence of genetic factors in congenic B10 and BALB mice of H-2q, H-2k, and H-2b haplotypes was examined following oral infection with a low-virulence strain. Whereas B10 mice were highly susceptible, BALB mice had a less severe and more protracted disease. Within the two congenic groups, the major histocompatibility complex haplotype had a strong impact on the disease. The H-2k haplotype was associated with early death in B10 mice but with a favourable outcome in BALB mice, whereas the reverse was observed for the H-2q haplotype. These findings indicate that genetically determined factors are critically involved in determining the intracerebral immune response and the course of murine toxoplasmosis, but significant differences between B10 and BALB mice point to a modulating role of additional genetic loci. Moreover, this genetic control of resistance against acute infection depends on the strain of *Toxoplasma gondii.*[18] The use of transgenic mouse models has shown that human MHC genes had a role on the number of brain cysts. Introduction of HLA-B27 into B10 mice made them more susceptible to cyst formation.[14] Interestingly, recent preliminary results have shown that in infants with severe congenital toxoplasmosis the frequency of HLA class II allele DQ3 was increased though the frequency of this allele in the mother did not differ from that in the normal population.[4] In addition, marked differences in infection patterns have been observed between dizygotic twins compared to the similar patterns between monozygotic twins.[14]

### Sex Susceptibility

Female mice were found to be more susceptible to acute infection, as determined by higher mortality levels, than male mice. Female mice surviving chronic infections harboured more cysts in their brains than did surviving males. Spleen cells from male mice produced higher levels of interferon (INF)-gamma in the early stages of infection than those from female mice.[19] Male severe combined immunodeficiency (SCID) mice were also more resistant than female mice to infection with *Toxoplasma gondii* producing interleukin (IL)-12 more rapidly and exhibiting higher levels of INF-gamma.[20] There is no data concerning differential susceptibilities to toxoplasmic infections between human males and females.

### ARE THERE FACTORS FACILITATING TRANSPLACENTAL TRANSMISSION OF *TOXOPLASMA*?

A primary infection with *Toxoplasma gondii* will induce in the infected host a humoral and cellular immune response.

### Factors Involved in the Immune Response

The acute phase of the disease is characterised in humans and mammals by a significant increase of specific IgA and IgM antibodies directed against the main major antigenic component P30 or SAG1.[21] This SAG1 has an important role in the invasion of host cells: monoclonal antibodies directed against SAG1 will block invasion,[22] the chronic stage being characterised by the presence of specific IgG. However, these circulating antibodies, witnesses of the infection, are useless to control the disease. As with most intracellular parasites, this role should be set down to cellular immunity and particularly to T cells. INF-gamma has a crucial role against this infection: mice treated by anti-INF-gamma or deficient for INF-gamma gene will die of toxoplasmosis.[23,24] The first cells to be activated are natural killer (NK) and monocytic cells which will produce INF-gamma and IL-12. IL-12 induces the lymphocyte population (CD4+ and CD8+) to a Th1 phenotype inhibiting *Toxoplasma* growth. The Th2 derived cytokines will have a counter-regulatory effect.[25]

### Influence of Pregnancy on the Immune Response

Some authors have recently shown that human placenta was escaping the maternal immune system by releasing immunosuppressive cytokines such as IL-4, IL-6 and IL-10.[26] This pattern of cytokine secretion, characteristic of a relative increase in Th2-associated immunity and decreased Th1 immunity, could facilitate the survival and/or the passage of the parasite through the placenta, but this point remains unproven for *Toxoplasma.* Candolfi et al. orally infected BALB/c mice and showed that pregnant mice had a larger lung parasite load 7 days post infection and a significantly higher number of brain cysts 30 days post infection.[27] The different patterns of cytokines described failed to explain this increase susceptibility, though high titers of the immunosuppressive IL-10 were observed. Hohlfeld et al. reported in 1990[28] the different subsets of T cells observed in infected pregnant women and their fetuses. Women infected by *Toxoplasma* during their pregnancy showed an increase of CD8 suppressor T cells and a decrease of CD4 helper T cells. These alterations were more important when the fetus was infected, suggesting that transmission could be enhanced by an impaired T cell immunity secondary to pregnancy. Interestingly, children with congenital toxoplasmosis have a specific immunological tolerance for *Toxoplasma gondii* with a selective reduction of INF-gamma production.[25]

### ARE ANIMAL MODELS USEFUL TO UNDERSTAND PATHOGENESIS OF HUMAN TOXOPLASMOSIS DURING PREGNANCY?

The human placenta structure (hemochorial) is similar to the placental structure of mice, rats, rabbits and monkeys (but different from the placental structure of dogs, cats, sheep, and pigs). Therefore, a lot of information could have been obtained from easy to handle experimental animals, but, curiously, the number of papers describing models of congenital toxoplasmosis are relatively few.

### Rodent Models

Older studies with outbred mice showed that transmission was possible through successive generations. However, studies conducted by Roberts et al.[15] with BALC/c mice demonstrated that transmission occurred only in mice infected for the first time during pregnancy. In an experimental model using orally infected Fisher rats, transmission to fetuses was only possible in rats infected for the first time.[12] These results were obtained with two different strains of *Toxoplasma* and with different modes of infection (orally and intraperitoneally).

### Primate Model

Schoondermark-Van de Ven described a very interesting rhesus monkey model of *Toxoplasma* congenital infection. Transmission to the fetus was investigated after maternal infection at day 90 or day 130 of pregnancy. A parasitemia was induced and lasted for about 10 days as proven by mouse inoculation and nested polymerase chain reaction (PCR) on blood samples. An overall transmission rate of 61% was found; this rate is similar to that found in humans.[29] A lot of interesting results were obtained on the efficacy of treatments usually prescribed in humans. Spiramycin accumulated in the soft tissues, especially in the liver and spleen, of both the mother and the fetus and was concentrated in placental tissue and in amniotic fluid.[30] In four monkeys that received treatment for about 7 weeks, the parasite was not present at birth in the placenta nor in amniotic fluid or neonatal organs.[31] Pyrimethamine and sulfadiazine was administered to six monkeys, in whose fetuses infection was diagnosed antenatally. The parasite was no longer detectable in the next consecutive amniotic fluid sample, taken 10 to 13 days after treatment was started. Furthermore, *Toxoplasma gondii* was not found in the neonate at birth. The parasite was still present at birth in three of four untreated fetuses that served as controls.[32] Immune responses (beside circulating antibodies) were not studied in these experiments.

### Absence of In Vitro Models

Important data could be obtained from in vitro models similar to what was described for malaria.[33] Cultivation of human synctiotrophoblast with *Toxoplasma* would permit the study of molecules mediating attachment and their expression in presence of different cytokines and hormones. An upregulation of adhesion molecules has been described in the pathogenesis of murine toxoplasmic encephalitis.[34]

### CAN A SEROPOSITIVE HOST TRANSMIT *TOXOPLASMA* TO THE FETUS?

This important point has been already discussed previously in rodents where older data reported that vertical transmission through successive generations was the normal situation in outbred mice.[15] The dogma for humans (and ovids) is that only infection for the first time during pregnancy results in congenital infections. But this dogma could be run down by recent observations. Desmonts et al.[35] reported five cases of congenital toxoplasmosis consecutive to a maternal *Toxoplasma* infection that had preceded pregnancy. One woman with a normal immune system had developed a well-documented lymph node toxoplasmosis 2 months before conceiving. Four women had chronic toxoplasmosis diagnosed in the course of an immunosuppressive disease: Hodgkin's disease in one case, systemic lupus erythematosus in two cases and pancytopenia in one case. Toxoplasmosis had been recognised 3, 5 and 10 years respectively before conception in three women, and at an uncertain date in one woman. Three women had received corticosteroids during pregnancy, and two had undergone splenectomy. Among the six children (two were twins), one presented with severe fetal disease at birth, one developed lethal systemic toxoplasmosis after birth, one showed hydrocephalus with therapeutically well-controlled chorioretinitis, one had an isolated eye lesion and two had asymptomatic infection. The parasite seems to have been transmitted after the 20th week of pregnancy in all cases. Pons et al.[36] and Vogel et al.[37] reported a few years later two additional woman with normal immune systems who developed toxoplasmosis 2 months before conceiving and who transmitted the disease to their fetuses. Though Marty et al.[38] reported a case of severe fetal toxoplasmosis resulting of toxoplasmic reactivation in an human immunodeficiency virus (HIV)-1 seropositive woman, this phenomenon appears very uncommon as proven by subsequent studies in cohorts of HIV-positive pregnant women.[39,40]

### Reinfection During Pregnancy

More disturbing is the case we have recently reported.[41] This case of congenital transmission was revealed by macular chorioretinitis in a nine-month-old child and confirmed by a retrospective serological analysis of the mother's sera. This woman tested positive for anti-*Toxoplasma* antibodies at 11 weeks of amenorrhoea (moderate titers of IgG antibodies without IgM, thus reflecting an old infection). No further toxoplasmic surveillance was therefore carried out during pregnancy. At delivery, clinical examination of the newborn was normal. However, 9 months later, the child developed divergent strabismus. Ophthalmologic examination revealed a macular chorioretinitis scar, with loss of right-sided vision. Congenital toxoplasmosis was confirmed by the detection of IgM antibodies in the child's serum. As the mother took part in a hematological survey during pregnancy, stored sera were available for analysis. Reinfection during the pregnancy was strongly suggested by the emergence of IgM and IgA antibodies and a rise in IgG titers between 11 and 28 W.A. The maternal reinfection was probably linked to oocysts, as her personal diary referred to contacts with kittens on week 18 of amenorrhoea, and to a flu-like illness a week later. There were no iatrogenic or apparent pathological factors of immunodeficiency, or abnormalities in lymphocyte subsets. To our knowledge, two other similar case have been reported.[42,43] Reinfection of apparently immunocompetent women during pregnancy is fortunately uncommon, but raises the possibility that ingestion of *Toxoplasma* cysts contained in meat does not protect against reinfection by oocysts. This could be explained by the antigenic differences between sporozoites and tachyzoites described by Kasper and Ware[5] but also by strain differences. Recently Araujo et al. published evidences that chronic infection in SW mice with the ME49 *Toxoplasma gondii* strain did not prevent acute disease or colonisation of the brain after reinfection with cysts of a variant of the C56 strain resistant to atovaquone.[44]

### CONCLUSION

The immunopathogenesis of toxoplasmosis during pregnancy is not completely understood. However, most published data suggest a role of the genetic background of the host and of the parasite. Models of congenital toxoplasmosis have been evaluated, but it appears that the conclusion drawn would be barely appropriate to understand the pathogenesis in pregnant women. A lot of trials (not reviewed here) have been performed to evaluate *Toxoplasma* proteins which could be used as vaccines. Most trials were performed with the P30 protein (SAG1) but had limited efficiency. In France, a national survey has recently shown a 54.3% prevalence of *Toxoplasma* antibodies in pregnant women, and the incidence of seroconversion in nonimmune women at the beginning of pregnancy was of 1.5%.[45] In France, a national prevention program was set up in the late seventies requiring compulsory serology before wedding and during the first trimester of pregnancy. If the serology is negative at the beginning of pregnancy, hygienic recommendations will be prescribed and the woman's serum should be checked every 3–4 weeks in order to detect a toxoplasmic infection very early. In case of a seroconversion, the fetus will be assessed regularly by echography and a prenatal diagnosis will be performed at 20–22 weeks of pregnancy. This French national prevention program could prevent 700 to 2650 cases per year of congenital toxoplasmosis with an annual cost of $30 to 60 million.

### REFERENCES

1. Dupouy-Camet J, Gavinet MF, Paugam A, Tourte-Schaefer C: Mode de contamination, incidence et prévalence de la toxoplasmose. Med Mal Infect 23S:139–147, 1993.
2. Chatterton JMW: Pregnancy. In: Ho-Yen DO, Joss AWL (eds.): Human toxoplasmosis. Oxford: Oxford University Press, pp 144–183, 1992.
3. Wong SY, Remington JS: Toxoplasmosis in pregnancy. Clin Infect Dis 18: 853–862, 1994.
4. Smith JE, Boothroyd JC, Hunter C, Petersen E: Progress in toxoplasmosis research. Parasitol Today 13:245–247, 1997.
5. Kasper LH, Ware PL: Recognition and characterisation of stage-specific oocyst/sporozoite antigens of *Toxoplasma gondii* by human antisera. J Clin Invest 75:1570–1577, 1985.
6. Kasper LH: Identification of stage-specific antigens of *Toxoplasma gondii*. Infect Immun 57: 668–672, 1989.
7. Dardé ML, Bouteille B, Pestre-Alexandre M: Isoenzyme analysis of 35 *Toxoplasma gondii* isolates and the biological and epidemiological implications. J Parasitol 78:786–794, 1992.
8. Dardé ML: Biodiversity in *Toxoplasma gondii*. In: Gross U (ed.): Current Topics in Microbiology and Immunology. Vol. 219: *Toxoplasma gondii*. Berlin: Springer, pp. 27–41, 1996.
9. Howe DK, Honore S, Derouin F, Sibley LD: Determination of genotypes of *Toxoplasma gondii* strains isolated from patients with toxoplasmosis. J Clin Microbiol 35: 1411–1414, 1997.
10. Howe DK, Summers BC, Sibley LD:Acute virulence in mice is associated with markers on chromosome VIII in *Toxoplasma gondii*. Infect Immun 64:5193–5198, 1996.
11. Guo ZG, Gross U, Johnson AM: *Toxoplasma gondii* virulence markers identified by random amplified polymorphic DNA polymerase chain reaction.Parasitol Res 83: 458–463, 1997.
12. Zenner L, Darcy F, Cesbron-Delauw MF, Capron A: Rat model of congenital toxoplasmosis: rate of transmission of three *Toxoplasma gondii* strains to fetuses and protective effect of a chronic infection. Infect Immun 61:360–363, 1993.
13. McLeod R, Johnson J, Estes R, Mack D: Immunogenetics in pathogenesis of and protection against toxoplasmosis. In: Gross U (ed.): Current Topics in Microbiology and Immunology. Vol 219: *Toxoplasma gondii*. Berlin: Springer, pp. 95–112, 1996.
14. McLeod R, Skamene E, Brown CR, Eisenhauer PB, Mack DG: Genetic regulation of early survival and cyst number after peroral *Toxoplasma gondii* infection of A × B/B × A recombinant inbred and B10 congenic mice. J Immunol 143:3031–3034, 1989.
15. Roberts CW, Alexander J: Studies on a murine model of congenital toxoplasmosis: vertical disease transmission only occurs in BALB/c mice infected for the first time during pregnancy. Parasitol 104:19–23, 1992.
16. Brown CR, McLeod R: Class I MHC genes and CD8+ T cells determine cyst number in *Toxoplasma gondii* infection. J Immunol 145:3438–3441, 1990.
17. Deckert-Schluter M, Schluter D, Schmidt D, Schwendemann G, Wiestler OD, Hof H: *Toxoplasma* encephalitis in congenic B10 and BALB mice: impact of genetic factors on the immune response. Infect Immun 62:221–228,1994.
18. Suzuki Y, Yang Q, Remington JS: Genetic resistance against acute toxoplasmosis depends on the strain of *Toxoplasma gondii*. J Parasitol 81:1032–1034, 1995.
19. Roberts CW, Cruickshank SM, Alexander J: Sex-determined resistance to *Toxoplasma gondii* is associated with temporal differences in cytokine production. Infect Immun 63:2549–2555, 1995.
20. Walker W, Roberts CW, Ferguson DJ, Jebbari H, Alexander J: Innate immunity to *Toxoplasma gondii* is influenced by gender and is associated with differences in interleukin-12 and gamma interferon production. Infect Immun 65:1119–1121, 1997.
21. Joss AWL. 1995. Diagnosis In: Ho-Yen DO, Joss AWL (eds.): Human toxoplasmosis. Oxford: Oxford University Press, pp. 79–118, 1992.
22. Grimwood J, Smith JE: *Toxoplasma gondii:* the role of parasite surface and secreted proteins in host cell invasion. Int J Parasitol 26:169–173, 1996.
23. Suzuki Y, Orellana MA, Schreiber RD, Remington JS: Interferon-gamma: the major mediator of resistance against *Toxoplasma gondii*. Science 240:516–518, 1988.
24. Sher A, Denkers EY, Gazzinelli RT: Induction and regulation of host cell-mediated immunity by *Toxoplasma gondii*. Ciba Found Symp 195:95–104, 1995.
25. Gomez Marin JE, Pinon JM, Bonhomme A, Guenounou M: Does human toxoplasmosis involve an imbalance in T1/T2 cytokines. Med Hyp 48:161–169, 1997.
26. Formby B: Immunologic response in pregnancy. Its role in endocrine disorders of pregnancy and influence on the course of maternal autoimmune disease. Endocrinol Met Clin North Am 24:187–205, 1995.

27. Hohlfeld P, Forestier F, Marion S, Thulliez P, Marcon P, Daffos F: *Toxoplasma gondii* infection during pregnancy: T lymphocyte sub populations in mothers and fetuses. Pediatr Infect Dis J 9:878–81, 1990.
28. Candolfi E, Villard O, Thouvenin M, Kien TT: Role of NO-induced immune suppression in toxoplasmosis during pregnancy and in infection by a virulent strain of *Toxoplasma gondii.* In: Gross U (ed.): Current Topics in Microbiology and Immunology. Vol 219. *Toxoplasma gondii.* Berlin: Springer, pp. 141–154, 1996.
29. Schoondermark-Van de Ven E, Melchers W, Galama J, Camps W, Eskes T, Meuwissen J: Congenital toxoplasmosis: an experimental study in rhesus monkeys for transmission and prenatal diagnosis. Exp Parasitol 77: 200–211, 1993.
30. Schoondermark-Van de Ven E, Galama J, Camps W, et al.: Pharmacokinetics of spiramycin in the rhesus monkey: transplacental passage and distribution in tissue in the fetus. Antimicrob Agents Chemother 38:1922–1929, 1994.
31. Schoondermark-Van de Ven E, Melchers W, Camps W, Eskes T, Meuwissen J, Galama J: Effectiveness of spiramycin for treatment of congenital *Toxoplasma gondii* infection in rhesus monkeys. Antimicrob Agents Chemother 38:1930–1936, 1994.
32. Schoondermark-van de Ven E, Galama J, Vree T, et al.: Study of treatment of congenital *Toxoplasma gondii* infection in rhesus monkeys with pyrimethamine and sulfadiazine. Antimicrob Agents Chemother 39:137–144, 1995.
33. Maubert B, Guilbert LJ, Deloron P: Cytoadherence of Plasmodium falciparum to intercellular adhesion molecule 1 and chondroitin-4-sulfate expressed by the syncytiotrophoblast in the human placenta. Infect Immun 65:1251–1257, 1997.
34. Deckert-Schluter M, Schluter D, Hof H, Wiestler OD, Lassmann H: Differential expression of ICAM-1, VCAM-1 and their ligands LFA-1, Mac-1,CD43, VLA-4, and MHC class II antigens in murine *Toxoplasma* encephalitis: a light microscopic and ultrastructural immunohistochemical study. J Neuropathol Exp Neurol 53:457–468, 1994.
35. Desmonts G, Couvreur J, Thulliez P: Toxoplasmose congénitale: Cinq cas de transmission à l'enfant d'une infection maternelle antérieure à la grossesse. Presse Med 19:1445–1449, 1990.
36. Pons JC, Sigrand C, Grangeot-Keros L, Frydman R, Thulliez P: Toxoplasmose congénitale: transmission au foetus d'une infection maternelle antéconceptionnelle. Presse Med 24:179–182, 1995.
37. Vogel N, Kirisits M, Michael E, et al.: Congenital toxoplasmosis transmitted from an immunologically competent mother infected before conception. Clin Infect Dis 23:1055–1060, 1996.
38. Marty P, Bongain A, Rahal A, et al.: Prenatal diagnosis of severe fetal toxoplasmosis as a result of toxoplasmic reactivation in an HIV-1 seropositive woman. Prenat Diagn 14:414–415, 1994.
39. Minkoff H, Remington JS, Holman S, Ramirez R, Goodwin S, Landesman S: Vertical transmission of *Toxoplasma* by human immunodeficiency virus-infected women. Am J Obstet Gynecol 176: 555–559, 1997.
40. European Collaborative Study and Research Network on Congenital Toxoplasmosis: Low incidence of congenital toxoplasmosis in children born to women infected with human immunodeficiency virus. Eur J Obstet Gynecol Reprod Biol 68: 93–96,1996.
41. Gavinet MF, Robert F, Firtion G, et al.: Congenital toxoplasmosis due to maternal reinfection during pregnancy. J Clin Microbiol 35: 1276–1277, 1997.
42. Fortier B, Aissi E., Ajana F, et al.: Spontaneous abortion and reinfection by *Toxoplasma gondii.* Lancet 338: 444, 1991.
43. Hennequin C, Dureau, N'Guyen L, Thulliez P, Gagelin B, Dufier JL: Congenital toxoplasmosis acquired from an immune woman. Ped Inf Dis J 16:75–77, 1997.
44. Araujo F, Slifer T, Kim S: Chronic infection with *Toxoplasma gondii* does not prevent acute disease or colonization of the brain with tissue cysts following reinfection with different strains of the parasite. J Parasitol 83: 521–522,1997.
45. Ancelle T, Goulet V, Tirard-Fleury V, et al.: La toxoplasmose chez la femme enceinte en France en 1995. Résultat d'une enquête nationale périnatale. BEH 51: 227–229, 1996.
